# From Prevention to Management: Understanding Postoperative Infections in Gynaecology

**DOI:** 10.7759/cureus.46319

**Published:** 2023-10-01

**Authors:** Vaishnavi Ronghe, Anushree Modak, Kavita Gomase, Manjusha G Mahakalkar

**Affiliations:** 1 Obstetrics and Gynaecology, Smt. Radhikabai Meghe Memorial College of Nursing, Datta Meghe Institute of Higher Education & Research, Wardha, IND

**Keywords:** multidisciplinary collaboration, emerging trends, management, prevention, gynecology, postoperative infections

## Abstract

This narrative review examines the multifaceted realm of postoperative infections in gynaecology, addressing their significance, types, risk factors, prevention, management, and emerging trends. Postoperative infections, encompassing surgical site infections, urinary tract infections, and pelvic inflammatory disease, pose considerable challenges in patient care, warranting comprehensive exploration. Strategies for prevention include preoperative patient assessment, antimicrobial prophylaxis, and aseptic techniques. Intraoperative measures encompass infection control and instrument sterilization, while postoperative care involves wound management and early infection detection. Diagnostic tools, including blood tests, imaging, and microbiological cultures, aid in timely identification. Management strategies encompass antibiotic therapy, surgical interventions, supportive care, and addressing complications. The review underscores the necessity of personalized approaches, multidisciplinary collaboration, and innovative technologies in future infection management. It calls for ongoing research, heightened awareness, and meticulous care to minimize the impact of postoperative infections and optimize patient outcomes.

## Introduction and background

Postoperative infections following gynecological surgeries pose a significant clinical challenge, impacting patient outcomes, healthcare costs, and overall quality of life. These infections lead to prolonged hospital stays and increased medical expenses and contribute to patient discomfort, delayed recovery, and potential long-term complications. Addressing postoperative infections is paramount in modern healthcare, as it aligns with improving patient safety, enhancing surgical success rates, and optimizing healthcare resource allocation [1].

Gynecological surgeries encompass various procedures, including hysterectomies, cesarean sections, laparoscopic interventions, and various reproductive health treatments. Each of these procedures carries a distinct risk of postoperative infections due to factors such as surgical site contamination, compromised immune responses, and the presence of foreign bodies. Recognizing and understanding the nuances of postoperative infections in gynecology is crucial for healthcare providers to develop effective preventive strategies and management approaches [2].

Postoperative infections can manifest as surgical site infections (SSIs), urinary tract infections (UTIs), and pelvic inflammatory disease (PID). SSIs involve infections at the surgical site, either superficial or deep, and may lead to cellulitis, abscess formation, or organ involvement. UTIs can arise due to catheterization during surgery or other procedural interventions, while PID often results from ascending infections involving the reproductive organs. These infections are frequently caused by bacteria such as *Staphylococcus aureus*, *Escherichia coli*, and various anaerobes [1].

The incidence of postoperative infections varies depending on the type of procedure, patient characteristics, and adherence to infection prevention protocols. The introduction of minimally invasive techniques has significantly reduced the risk of infections in some cases. Still, challenges persist, warranting a comprehensive understanding of risk factors, prevention measures, and effective treatment strategies [3].

This narrative review aims to comprehensively analyze postoperative infections in gynaecology, ranging from their underlying causes and risk factors to strategies for prevention and optimal management. This review aims to equip healthcare practitioners, surgeons, and researchers with valuable insights to enhance patient care and outcomes by synthesizing current research, best practices, and emerging trends. By exploring the latest advancements in infection prevention, diagnostic techniques, and treatment modalities, we intend to contribute to the ongoing efforts to reduce the burden of postoperative infections in gynaecological surgeries.

## Review

Surgical site infections (SSIs)

Definition and Classification

Surgical site infections (SSIs) are a significant subset of postoperative infections in gynecology. These infections manifest at the site of surgical incisions and can be classified into distinct categories, each delineating the extent of tissue involvement and potential complications [4].

Superficial SSIs: Among the classifications, superficial SSIs are confined to the skin and subcutaneous layers surrounding the surgical incision. They manifest as redness, localized warmth, and tenderness around the wound area. While these infections generally pose a lower risk of severe complications, they can progress and potentially develop into more severe forms if left untreated [5].

Deep SSIs: Infections surpassing superficial layers and infiltrating deeper tissues, such as muscles, are categorized as deep SSIs. These infections often present with increasing pain, swelling, and sometimes purulent drainage from the wound. Deep SSIs pose a more significant concern, as they may lead to abscess formation, delayed wound healing, and prolonged recovery periods [6].

Organ or space SSIs: The most complex within this classification, organ or space SSIs involve any body cavity or organ manipulated or opened during surgery. For instance, abdominal or pelvic cavities could become sites of infection. The consequences of these infections can be particularly severe, potentially leading to intra-abdominal abscesses, sepsis, and a heightened risk of long-term complications [7].

Pathogens Commonly Involved

Surgical site infections (SSIs) represent a significant concern in postoperative complications. One critical aspect of understanding SSIs is recognising the range of pathogens that can contribute to their development. These pathogens originate from various sources, including the patient's skin flora, the surgical environment, and even the healthcare providers involved in the procedure [8]. When discussing the pathogens commonly involved in SSIs, it's important to note their classification based on their staining characteristics: Gram-positive and Gram-negative bacteria. This distinction is relevant because it provides insights into their structural and functional differences, which can influence their response to antibiotics and the body's immune system [9].

*Staphylococcus aureus*, a Gram-positive bacterium, is a well-known pathogen frequently associated with SSIs. Its ability to cause both superficial and deep infections is particularly concerning. Additionally, methicillin-resistant *Staphylococcus aureus* (MRSA) strains have emerged, which are resistant to many commonly used antibiotics, complicating treatment efforts and underscoring the need for vigilant infection control measures [10].

Streptococcus species are another group of Gram-positive bacteria that can contribute to SSIs. These bacteria, part of the normal human flora, can become opportunistic pathogens under certain conditions, leading to infections at the surgical site [11]. On the other hand, Gram-negative bacteria, such as *Escherichia coli* and other enteric bacteria, are also significant contributors to SSIs. These bacteria can access the surgical site from the gastrointestinal tract, especially in procedures involving the abdominal or pelvic regions [11].

Urinary tract infections (UTIs)

Urinary tract infections represent a notable postoperative complication, frequently arising from inserting urinary catheters during surgical procedures. UTIs encompass infections affecting various parts of the urinary system, which include the bladder, urethra, and kidneys. The urinary tract, responsible for eliminating waste and excess bodily fluids, is susceptible to colonization by bacteria introduced during catheterization [12].

Urinary catheters are sometimes employed to monitor urine output and maintain a sterile surgical field during surgical interventions. However, the insertion of indwelling urinary catheters can inadvertently introduce bacteria into the urinary tract, potentially leading to infection. The catheter acts as a conduit, allowing microbes to ascend into the bladder and beyond [12].

Urinary tract infections can manifest as a range of symptoms, including frequent urination, a strong urge to urinate, a burning sensation during urination, cloudy or bloody urine, and discomfort or pain in the lower abdomen. In more severe cases, UTIs can lead to complications such as pyelonephritis, an infection of the kidneys, which can cause high fever, back pain, and systemic symptoms [13].

Preventing UTIs in the postoperative setting involves employing aseptic techniques during catheter insertion and maintenance and minimizing the duration of catheterization. Healthcare providers should adhere to strict protocols to reduce the risk of catheter-associated infections. Furthermore, timely removal of the catheter once it is no longer medically necessary is crucial to prevent ongoing exposure to potential sources of infection [14].

Pelvic inflammatory disease (PID)

Pelvic Inflammatory Disease (PID) represents a significant postoperative infection with distinct characteristics and implications. This condition involves inflammation within the female reproductive organs, explicitly affecting critical structures such as the uterus, fallopian tubes, and ovaries. It's important to note that PID can have various causes, with sexually transmitted infections (STIs) being one of the primary culprits. However, it's not limited to sexually active individuals, as non-sexually transmitted pathogens can also contribute to its development [15].

Complex Nature and Causes

PID is considered complex due to its potential to involve multiple reproductive organs and its potential to stem from various sources. The causative agents, which include pathogens such as chlamydia and gonorrhoea, can be mentioned alongside other factors that contribute to PID. While sexual activity and the spread of STIs can trigger PID, it's also recognized that postoperative infections and ascending infections from other body parts can lead to its development. In gynaecological surgeries, surgical interventions can inadvertently introduce these pathogens into the reproductive tract, especially if proper aseptic techniques are not maintained [15].

Long-Term Consequences

One of the concerning aspects of PID is its long-term impact on women's health. The inflammation caused by PID can lead to scar tissue formation and adhesions in the reproductive organs. These adhesions can impair the normal functioning of the fallopian tubes, potentially leading to infertility or an increased risk of ectopic pregnancies. Additionally, PID can result in chronic pelvic pain, significantly affecting a woman's quality of life. The potential for chronic pain and fertility issues underscores the importance of prevention and effective PID management [15].

Diagnosis and Treatment

Diagnosing PID often involves clinical assessment, laboratory tests, and imaging. Patients may present with lower abdominal pain, abnormal vaginal discharge, painful urination, and fever. Diagnostic tests may include pelvic exams, blood tests to check for signs of infection, and imaging studies like ultrasounds or MRIs to visualize the reproductive organs. Early detection and timely treatment are crucial to prevent complications. Treatment typically involves a course of antibiotics to target the underlying infection. Hospitalization and more aggressive interventions may be necessary in severe cases or abscess formation [15].

Prevention and Patient Education

Preventing PID requires a comprehensive approach, particularly in the context of gynaecological surgeries. Ensuring proper aseptic techniques during surgery, minimizing the introduction of pathogens, and promptly treating any infections that do occur are vital. Additionally, patient education is critical. Informing patients about the risks of PID, especially after surgical procedures, empowers them to recognize symptoms early and seek timely medical attention, ultimately aiding in better outcomes and reducing long-term complications [16].

Risk factors for postoperative infections

Patient-Related Factors

Patient-related factors are integral contributors to an individual's susceptibility to postoperative infections. These factors encompass a spectrum of characteristics that influence the body's ability to defend against infections and heal after surgery. Understanding these factors aids healthcare providers in tailoring preventive strategies and optimizing patient care. Here are some critical patient-related factors and their roles in determining infection susceptibility.

Age: Age is a significant determinant of infection risk. The very young and the elderly face heightened vulnerability to postoperative infections due to variations in their immune responses. Infants and young children possess developing immune systems that may not offer robust protection against infections. Conversely, elderly individuals often experience a decline in immune function, rendering them less capable of effectively fighting off pathogens. As a result, infections can more easily take hold and lead to complications in these age groups [17].

Obesity: Obesity poses a multifaceted challenge in postoperative infection susceptibility. Conversely, poor nutritional status, particularly in the context of protein-energy malnutrition (PEM), also significantly impacts the healing process, especially in cancer patients. In obese individuals, increased adipose tissue can hinder the optimal wound-healing process by creating an environment conducive to bacterial growth. This excess tissue may impede the body's ability to close and heal surgical incisions effectively. Additionally, obesity is often associated with comorbidities such as diabetes and cardiovascular disease, further elevating the risk of infection [18]. It is crucial to recognize that both obesity and poor nutritional status can significantly influence the postoperative healing trajectory.

Immune status: Patients with compromised immune systems, such as diabetes, autoimmune disorders, or undergoing immunosuppressive treatments, are inherently more susceptible to infections. Immune-compromised individuals have reduced defence mechanisms against pathogens, making it easier for infections to take hold and progress. Consequently, healthcare providers must take special precautions to minimize infection risks in these patients, including tailored antimicrobial prophylaxis and vigilant postoperative monitoring [19].

Underlying health conditions: Besides the chronic health conditions discussed earlier, it is imperative to consider other significant patient-related factors that can influence postoperative outcomes. These factors encompass a range of lifestyle and medical elements, and their impact on the healing process should not be underestimated. Smoking is one such factor; it can harm the body's ability to combat infections and impede healing, resulting in a higher risk of postoperative infections [20]. Patients with a history of alcohol or substance abuse face unique challenges in postoperative recovery, as the abuse of these substances can weaken the body's defences and complicate the healing process. Furthermore, patients with implanted medical devices, such as prosthetics or artificial joints, warrant special attention, as these foreign materials introduce additional considerations for postoperative care and the potential risk of infections or complications. When present in patients undergoing surgery, these factors necessitate a tailored approach to care and a heightened awareness of the potential challenges they pose to the healing process. Addressing these patient-related factors comprehensively is essential for optimizing surgical outcomes and minimizing the risk of complications [20].

Surgical Factors

The influence of surgical factors on the risk of postoperative infections is paramount, as they significantly affect patients' susceptibility to infections following surgical interventions. These factors encompass various elements within the surgical process, encompassing the surgical technique and considerations related to the surgical environment, the presence of foreign materials, and other critical factors [21].

Surgical technique: The choice of surgical technique plays a pivotal role in determining the risk of postoperative infections. Inherent in this decision is the size and nature of the incisions made during surgery. Open surgical procedures, characterized by larger incisions, inherently create a larger wound area, potentially elevating the risk of contamination and subsequent infection. Conversely, minimally invasive procedures like laparoscopic or robotic surgeries feature smaller incisions that reduce tissue trauma. This reduction in tissue exposure decreases the risk of infection by minimizing potential entry points for pathogens [21].

Duration of surgery: The duration of a surgical procedure is intrinsically linked to the potential risk of infection. Prolonged surgeries expose patients to pathogens for an extended duration, compromising tissue perfusion. Extended exposure to surgical instruments, equipment, and the surgical environment increases the likelihood of bacterial contamination and subsequent infection development [8].

Implantation of foreign bodies: Introducing surgical implants, devices, or foreign materials adds dimension to the risk of infection. These foreign bodies present surfaces where bacteria can adhere and form biofilms, posing a challenge to the immune system's ability to combat infection. The presence of implants, such as mesh in hernia repair or joint replacements, establishes a distinct environment that necessitates meticulous attention to prevent infections [22].

Contamination and sterilization: The proper sterilization of surgical instruments and maintaining a sterile surgical environment are critical in preventing postoperative infections. Inadequate sterilization or contamination of surgical instruments creates a direct pathway for transmitting pathogens into the patient's body. Strict adherence to rigorous sterilization protocols, thorough instrument cleaning, and strict observance of infection control practices are imperative in mitigating this risk [23].

Blood transfusions: It's important to note that blood transfusions during surgery may introduce an additional layer of infection risk if not rigorously screened and managed. Improperly screened or managed blood transfusions can introduce infections into the patient's system. Therefore, the vigilance of healthcare providers in ensuring the safety of blood transfusion practices is essential to minimize this risk [22].

Emergency surgery: Surgical procedures performed as emergencies, instead of elective surgeries, may carry a heightened risk of infections. The urgency of emergency surgery often allows for less preoperative preparation, leading to a higher risk of infections due to limited time for comprehensive sterilization and preparation procedures [23]. 

Healthcare-Associated Factors

Healthcare-associated factors play a pivotal role in developing and preventing postoperative infections. These factors are closely tied to the practices and conditions within the healthcare facility, which can significantly influence the risk of infections following surgical procedures. Addressing these factors is vital for ensuring patient safety and maintaining high standards of healthcare quality.

Infection control practices: Stringent adherence to infection control practices is a cornerstone in preventing healthcare-associated infections. This includes rigorous implementation of hand hygiene protocols, aseptic techniques, and sterile procedures. Regular and thorough handwashing by healthcare personnel is crucial for minimizing the transmission of potential pathogens from person to person. Aseptic techniques ensure that surgical environments and procedures are kept as free from contamination as possible, reducing the risk of introducing harmful microorganisms during surgery. Sterile procedures use sterile gloves, gowns, drapes, and instruments to maintain a sterile field and minimize the risk of introducing pathogens into the patient's body [24].

Antimicrobial prophylaxis: The proper administration of antibiotics before surgery, known as antimicrobial prophylaxis, is a critical preventive measure against postoperative infections. Administering the right antibiotics at the correct time helps to target potential pathogens that may be introduced during surgery. By reducing the microbial load before the procedure begins, antimicrobial prophylaxis significantly lowers the risk of infection development. However, it's essential to adhere to evidence-based guidelines for antibiotic use to avoid the development of antibiotic resistance [25].

Hospital environment: The hospital environment can contribute to the risk of infections, particularly hospital-acquired infections. Patients undergoing surgery are vulnerable to pathogens from other infected patients or contaminated surfaces in the hospital environment. Effective cleaning and disinfection of surfaces, equipment, and patient care areas are imperative to minimise the potential for cross-contamination. Isolating patients with known infections and implementing strict infection control protocols can help contain the spread of pathogens within the hospital environment [26].

Prevention of postoperative infections: preoperative measures

Patient Awareness and Optimization

Thorough patient assessment stands as a cornerstone in the endeavour to prevent postoperative infections. This critical step requires healthcare providers to meticulously evaluate patients regarding their immediate surgical needs and with a comprehensive understanding of their health profile. This involves delving into a patient's medical history, current health status, and potential risk factors [27].

One of the critical objectives of patient assessment is to identify any underlying health conditions that could increase the susceptibility to infections. For instance, chronic illnesses such as diabetes, hypertension, or autoimmune disorders can compromise the immune system's ability to ward off infections, necessitating tailored preventive strategies. Additionally, immunocompromised states, whether due to medical treatments or other factors, need to be recognized and managed adequately to mitigate the risk of infections [28].

Furthermore, the evaluation of risk factors associated with infections is paramount. Patient-specific factors, such as age, weight, and overall physical condition, significantly determine the likelihood of infections. For instance, elderly patients and those with a higher body mass index might have altered wound-healing processes, making them more prone to postoperative infections. Identifying these factors allows healthcare providers to design a proactive infection prevention plan [29].

Optimizing a patient's overall health before surgery is crucial in infection prevention. This goes beyond addressing immediate surgical concerns; it entails managing chronic conditions effectively, optimizing nutritional status, and addressing pre-existing infections. For example, managing diabetes through appropriate glycemic control not only aids in successful surgery but also reduces the risk of wound-healing complications and subsequent infections [27].

Patient assessment and optimization form a holistic strategy involving meticulous evaluation, identification of vulnerabilities, and tailoring interventions to individual patients. By recognizing and addressing factors that can compromise the body's defences against infections, healthcare providers can proactively enhance the patient's ability to withstand surgical stress, minimize the risk of postoperative infections, and foster improved overall outcomes [30].

Antimicrobial Prophylaxis Guidelines

Antimicrobial prophylaxis is a strategic approach in healthcare that entails the administration of antibiotics before surgery to minimize the risk of postoperative infections. This preventive measure is especially critical when surgical procedures could potentially introduce pathogens into the body. The rationale behind antimicrobial prophylaxis is to ensure that therapeutic levels of antibiotics are present in the body before and during the surgical procedure, thereby suppressing the growth of bacteria that could lead to infections [31].

The implementation of antimicrobial prophylaxis is guided by evidence-based protocols, which dictate the selection of appropriate antibiotics and their precise administration timing. These protocols are formulated based on a combination of factors, including the type of surgery, the patient's medical history, the potential pathogens involved, and the expected duration of the procedure. The goal is to ensure the antibiotics are effective against the likely causative agents while minimizing the risk of adverse effects [32].

This practice becomes particularly indispensable in surgeries that involve the implantation of foreign materials, such as prosthetics or medical devices, or when surgical interventions breach sterile body cavities. In these scenarios, the risk of infection is heightened due to the introduction of potential pathogens from external sources. Antimicrobial prophylaxis acts as a barrier, hindering the proliferation of bacteria at the surgical site and reducing the likelihood of infections taking hold [33].

It's important to underscore that while antimicrobial prophylaxis is crucial for infection prevention, the judicious use of antibiotics is paramount. Overuse or inappropriate administration of antibiotics can lead to unintended consequences, such as the development of antibiotic-resistant bacteria. To counteract this, healthcare providers adhere closely to established guidelines, considering factors such as dosage, timing, and duration of antibiotic administration. The overarching aim is to strike a balance between infection prevention and antibiotic stewardship, safeguarding the efficacy of these medications for future use [34].

Surgical Site Preparation

Proper surgical site preparation is critical in preventing contamination and reducing the risk of postoperative infections. The surgical site, where incisions are made to access the body's internal structures, is vulnerable to introducing bacteria and other microorganisms. Ensuring a sterile environment during surgery is essential to minimize the risk of infections [8].

Thorough cleansing and disinfection of the skin: Surgical site preparation involves thoroughly cleansing and disinfecting the patient's skin in and around the surgical area. This is typically done using antiseptic solutions that kill or inhibit the growth of microorganisms. The choice of antiseptic solution can vary, but commonly used solutions include chlorhexidine and iodine-based products [35].

Importance of Standardized Protocol

Consistency and strict adherence to a standardized protocol for surgical site preparation play a pivotal role in upholding aseptic conditions during surgical procedures. This protocol delineates a set of meticulous steps that the surgical team must meticulously follow before the commencement of the surgery. It is designed to ensure the highest level of cleanliness and minimize the risk of infections. This protocol comprises several crucial components that collectively contribute to creating an environment that is free from contaminants.

Hand hygiene: One of the fundamental steps is that all surgical team members must rigorously perform proper hand hygiene using antiseptic soap or hand sanitizer. This initial measure is paramount as it aims to eliminate any transient microbes on the hands, which could be introduced into the surgical field [24].

Patient preparation: Patient preparation involves methodical cleaning and disinfection of the patient's skin surrounding the surgical site. This is executed systematically, commencing from the centre and proceeding outward. By adhering to this technique, a progressively sterile field is established, minimizing the possibility of introducing microorganisms from the periphery into the area of operation [36].

Use of sterile drapes: Sterile drapes are positioned meticulously around the surgical site, creating an impermeable barrier between the sterile surgical field and non-sterile surrounding areas. This is a physical shield to prevent potential contaminants, such as airborne particles, from infiltrating the sterile environment [37-38].

Application of antiseptic solution: An antiseptic solution is meticulously applied to the surgical site. This process is executed carefully to ensure thorough coverage and maximum antiseptic effectiveness. The antiseptic solution eradicates or significantly reduces the presence of microorganisms on the patient's skin, further enhancing the sterile conditions [23].

Prevention of postoperative infections: intraoperative measures

Aseptic Techniques and Infection Control

Maintaining a sterile surgical field is a cornerstone of infection prevention in surgical procedures. Aseptic techniques and infection control practices are meticulously employed to create an environment that minimizes the risk of introducing harmful microorganisms into the patient's body during surgery. This practice is fundamental in preventing postoperative infections, which can affect patients' health and recovery [39].

Proper hand hygiene: Surgical teams rigorously follow hand hygiene protocols before and during procedures. Thorough handwashing with antimicrobial soap and water or using alcohol-based hand sanitizers helps eliminate transient microorganisms from the hands of healthcare personnel [24].

Wearing sterile attire: Surgical personnel don sterile gowns, gloves, and other protective gear to prevent surgical field contamination. Sterile attire acts as a barrier, preventing the shedding of skin cells, hair, and potential pathogens from reaching the surgical site [1].

Sterile barrier creation: Creating a sterile field around the surgical site involves using sterile drapes and covers to isolate the area from non-sterile surroundings. This minimizes the risk of airborne contaminants settling on the patient or surgical instruments [40].

Minimizing pathogen introduction: Every effort minimizes contact or exchange between non-sterile and sterile areas. Surgical instruments and supplies that come into contact with the surgical site are either sterilized or properly disinfected to eliminate potential pathogens [27].

Reducing the risk of infections: Adherence to aseptic techniques and infection control measures significantly reduces the risk of infections at the surgical site. By limiting the introduction of microorganisms that could lead to infection, surgical teams enhance patient safety and contribute to successful surgical outcomes [8].

Interdisciplinary collaboration: Maintaining a sterile field requires the coordinated effort of the entire surgical team, from surgeons and nurses to technicians and anesthesiologists. Clear communication, standard operating procedures, and a shared commitment to infection control are essential components of this collaborative effort [41].

Surgical Instrument Sterilization

Ensuring surgical instruments and equipment sterility is fundamental to infection prevention in healthcare settings. The meticulous sterilization of instruments is essential to mitigate the risk of transmitting infectious agents from one patient to another during surgical procedures. Surgical instrument sterilization involves adhering to rigorous standards and guidelines to guarantee patient safety.

Methods of Sterilization

Several methods are employed for surgical instrument sterilization, each tailored to eliminate all microorganisms, including bacteria, viruses, and spores. These methods include:

Autoclaving: Autoclaves use high-pressure steam to achieve sterilization. The heat and pressure effectively kill microorganisms, and this method is widely considered one of the most reliable ways to sterilize instruments. Autoclaving is suitable for various instruments and materials [42].

Chemical disinfection: Chemical disinfection involves using specialized solutions to destroy or inactivate microorganisms. While more thorough than sterilization, chemical disinfection can be used for instruments that cannot withstand the high heat of autoclaving. However, it is typically employed for items that do not penetrate tissues [42].

Other validated methods: Depending on the type of instruments and materials, other validated methods may include dry heat, radiation, and ethylene oxide gas sterilization. Each method has advantages and limitations, and selection is based on factors such as instrument composition, intended use, and recommended guidelines [42].

Importance of Regular Maintenance and Protocols

Regular maintenance and calibration of autoclaves, chemical baths, and other sterilization equipment are necessary to guarantee consistent and reliable outcomes. Deviations in equipment performance could compromise the efficacy of the sterilization process and increase the risk of infections [43]. Adherence to standardized protocols is equally vital. Protocols dictate the correct sequence of steps for instrument preparation, packaging, loading into sterilization equipment, and subsequent handling. Following these protocols meticulously minimizes the potential for errors that could lead to incomplete sterilization or contamination of instruments.

Prevention of postoperative infections: postoperative measures

Wound Care and Dressing

Effective wound care and proper dressing are critical in preventing and managing postoperative infections. After gynaecological surgeries, surgical incisions serve as potential entry points for pathogens, making meticulous wound care an essential practice to minimize the risk of infections. This process involves regular monitoring of the incision site, prompt recognition of any signs of infection, and appropriate dressing changes performed strictly with sterile techniques [1].

Monitoring and detection: Regular postoperative assessments of the surgical incision site are essential to monitor its healing progress and identify deviations from the expected healing trajectory. Signs of infection may include increasing redness, swelling, warmth, tenderness, or abnormal discharge or pus around the wound. The early detection of these signs is crucial for timely intervention and preventing the progression of an infection [44].

Dressing involves removing the existing dressing and applying a new sterile dressing to the wound. These changes serve several purposes, including maintaining a clean environment around the wound, absorbing excess exudate, and protecting the wound from external contaminants. Proper technique during dressing changes is vital to avoid introducing bacteria or other pathogens into the wound site [45].

Choice of dressing material: The appropriate dressing material is essential to balance wound protection with optimal healing conditions. Dressings can vary in terms of their absorbency, moisture retention, and permeability to air. The choice of dressing should facilitate wound healing by maintaining an appropriate moisture level and temperature while preventing bacterial contamination [46].

Sterile techniques: Dressing changes should be performed using strict aseptic techniques to minimize the risk of introducing new pathogens. This involves proper hand hygiene, sterile gloves, and sterile instruments and supplies. The dressing should be secured well but not tight for proper circulation and healing [47].

Early Detection of Infections

Early detection of postoperative infections is critical to effective patient care and successful treatment outcomes. If left undetected or untreated, postoperative infections can lead to complications, prolonged recovery times, and negative impacts on patient well-being. Therefore, healthcare providers emphasize educating patients about the signs and symptoms of potential infections and establishing a robust follow-up system to monitor patients' progress after surgery [48-51].

Regular Postoperative Follow-Up Visits

Monitoring progress: Healthcare professionals diligently evaluate the patient's recovery journey throughout these follow-up visits. This includes a comprehensive assessment of various factors such as wound healing, mobility, and the patient's overall well-being. By closely monitoring these facets, healthcare providers gain insight into the recovery trajectory and potential deviations that could signify an underlying issue [52].

Clinical examination: Central to these appointments is a comprehensive clinical examination performed by healthcare providers. This meticulous evaluation systematically assesses the surgical site, neighbouring tissues, and the patient's overall physiological state. By conducting a thorough examination, healthcare professionals can promptly detect any unusual signs or symptoms that might indicate the presence of infection or other complications [53].

Addressing concerns: Follow-up visits offer patients a valuable platform to communicate any concerns or experiences they may have encountered since the surgery. Patient-reported symptoms, observations, or feelings of discomfort are crucial information contributing to a holistic understanding of recovery. By actively addressing these concerns, healthcare providers can collaborate with patients in devising appropriate interventions [54].

Early intervention: One of the primary advantages of these follow-up appointments is the ability to initiate early intervention if any indications of infection arise. If signs of infection are detected during the clinical examination or based on patient feedback, healthcare providers can swiftly implement targeted actions. This may involve adjusting the treatment regimen, such as modifying antibiotic prescriptions, refining wound care practices, or conducting further diagnostic tests to characterize the infection's nature [55].

Patient Education and Follow-Up

Patient education and follow-up are integral to postoperative care, significantly contributing to infection prevention and optimal recovery. Properly informing patients about infection prevention and self-care post-surgery is crucial in reducing the risk of complications and promoting positive outcomes. This process involves conveying essential information to patients regarding various aspects of their recovery journey [1].

Educating patients about infection prevention: Providing patients with clear and accessible information about infection prevention measures is paramount. This includes explaining the importance of maintaining proper hygiene, particularly around the surgical site, to minimize the risk of bacterial contamination. Patients should understand the significance of hand hygiene, wound cleanliness, and appropriate medication use [55].

Self-care post-surgery: Equipping patients with knowledge about self-care practices is empowering. Patients should be educated on how to care for their incision site, change dressings if necessary, and recognize any abnormal signs or symptoms. Understanding what constitutes a normal healing process versus indications of infection empowers patients to take proactive steps in their recovery [56].

Recognition of signs of potential infections: Educating patients about the signs and symptoms of potential infections is crucial for early intervention. Patients should be informed about common indicators such as increased pain, redness, swelling, warmth, and unusual discharge from the surgical site. Clear communication ensures that patients can promptly recognize and report any concerning changes to their healthcare providers [57].

Empowerment through clear communication: Effective patient education is a partnership between healthcare providers and patients. Clear and open communication fosters trust and empowers patients to participate in their recovery actively. Patients who understand the rationale behind infection prevention measures are more likely to adhere to recommended practices and report any deviations [58].

Promoting proactive healthcare-seeking behaviour: By educating patients about potential risks and complications, healthcare providers encourage patients to seek medical attention promptly if they suspect an infection. This proactive approach can prevent infections from worsening and expedite appropriate treatment [59].

Clinical presentation

Recognizing Signs and Symptoms of Postoperative Infections

Fever: A rise in body temperature, often indicated by fever, can be a telling sign of an underlying infection. Fever emerging within the days or weeks following surgery strongly indicates potential infection. The body's immune response to invading pathogens triggers an elevated temperature as it fights off the infection [60].

Localized pain: Heightened and increasing pain near the surgical site warrants close attention. The presence of pain, along with tenderness and discomfort, could be indicative of an ongoing infection. If these sensations intensify and are accompanied by other signs, infection becomes a likely concern [61].

Redness and swelling: Inflammation is a characteristic response of the body to infections. In the context of postoperative infections, redness and swelling around the incision site are frequent indicators of an ongoing infection. This visible sign often accompanies other symptoms and is a straightforward visual cue for healthcare providers [62].

Pus or discharge: The presence of pus or abnormal discharge from the surgical wound area is a clear and direct sign of an active infection. Plus, a viscous fluid containing dead white blood cells, bacteria, and tissue debris forms due to the body's immune response to infection. Its appearance in the wound area indicates infection [63].

Malaise: General feelings of unwellness, fatigue, and weakness, collectively known as malaise, can be early indicators of an underlying infection. At the same time, these symptoms are not specific to postoperative infections; their presence, particularly in combination with other signs, warrants further investigation [64].

Diagnostic tools and tests

Blood Tests (Complete Blood Count, Inflammatory Markers)

Blood tests are invaluable for diagnosing postoperative infections, providing crucial insights into the patient's immune response and the potential presence of infection [65]. A complete blood count (CBC) offers a snapshot of different blood cell types, focusing on white blood cells. An elevated white blood cell count, particularly neutrophils, indicates an activated immune response, often occurring in an infection. Moreover, inflammatory markers such as C-reactive protein (CRP) and erythrocyte sedimentation rate (ESR) indicate systemic inflammation [65]. In the context of infections, these markers tend to rise due to the body's defensive reaction against pathogens. Integrating these blood tests into clinical assessment enhances diagnostic accuracy by providing objective evidence of infection and guiding appropriate intervention [65].

*Imaging Techniques* *(Ultrasound*, *CT* *scan)*

Imaging techniques play a crucial role in diagnosing infections extending beyond the surgical site, such as deep or internal infections like abdominal infections. Among these techniques, ultrasound is a non-invasive modality that is valuable for identifying fluid collections, including abscesses, either near the surgical incision or within the body. Its real-time imaging capabilities are instrumental in pinpointing areas of concern, thereby guiding further diagnostic or therapeutic actions. Complementing ultrasound, computed tomography (CT) scans offer detailed cross-sectional images, enabling comprehensive assessments of deeper tissues and potential complications [66]. By facilitating the visualization of anatomical structures and the detection of abnormalities, imaging techniques significantly enhance the accuracy of infection diagnosis. This, in turn, aids in formulating precise treatment strategies and interventions [66].

Microbiological Cultures

Microbiological cultures are a cornerstone of infection diagnosis, playing a critical role in identifying the pathogens responsible for the infection. These cultures involve collecting samples from the infected site, such as wound swabs, fluid collections, or blood samples, and cultivating them in a controlled environment to promote bacterial growth. Once grown, the bacteria can be identified and tested for antibiotic sensitivities. This information is vital for tailoring antimicrobial therapy, ensuring the chosen antibiotics are effective against the identified pathogens. Microbiological cultures confirm the presence of infection and guide the selection of appropriate treatment, minimizing the risk of antibiotic resistance and optimizing patient care [67].

Management of postoperative infections

Antibiotic Therapy

Empiric vs. targeted therapy: Antibiotic therapy is a cornerstone in effectively managing postoperative infections. Physicians often initiate empiric antibiotic therapy when the specific causative pathogen is uncertain. This approach involves selecting antibiotics based on the likely organisms associated with the surgical procedure and considering local antimicrobial resistance patterns. Essentially, it's a calculated assumption designed to cover a broad spectrum of potential pathogens that could be responsible for the infection. This early intervention aims to halt the infection's progression until further information is available [68].

Once the results of microbial cultures are obtained, a shift from empiric therapy to targeted therapy may occur. Targeted therapy is tailored to the identified pathogen's susceptibility profile, ensuring a more precise and effective treatment strategy. This transition is crucial in minimizing unnecessary antibiotic use, reducing the risk of antibiotic resistance, and optimizing patient care. The accuracy of targeted therapy is contingent on reliable and timely culture results, which guide the selection of antibiotics most likely to effectively combat the specific infecting microorganism [68].

Selection of antibiotics based on pathogens: The selection of antibiotics hinges on several factors, including the type of infection and the identity of the causative pathogens. Initial treatment often involves broad-spectrum antibiotics capable of combating many bacteria. This approach is designed to tackle the infection while awaiting culture results swiftly. However, reliance on broad-spectrum antibiotics can contribute to the development of antibiotic resistance, which underscores the importance of transitioning to targeted therapy based on culture findings [69].

Culture results identify the microorganisms responsible for the infection and reveal their susceptibility to different antibiotics. With this information, healthcare providers can refine the treatment regimen, choosing effective antibiotics that minimize collateral damage to beneficial bacteria. In cases of antibiotic resistance, consultation with infectious disease specialists is valuable. These experts can offer insights into alternative antibiotic options, combination therapies, or even the exploration of newer antimicrobial agents [70].

Surgical interventions

Drainage of Abscesses or Collections

In postoperative infections where deep or organ/space surgical site infections (SSIs) result in the formation of abscesses or fluid collections, drainage emerges as a critical surgical intervention. Abscesses, which are pockets of pus or infected fluid, can develop in response to localized infection. By performing drainage, healthcare providers aim to achieve multiple objectives. First, it helps relieve pressure within the affected area, which can be essential for reducing pain and preventing further tissue damage. Second, removing infected material helps eliminate the nidus of infection, facilitating the body's natural healing processes. This procedure is essential as it can prevent the spread of infection to nearby structures. The precision of modern medical imaging, such as ultrasound or CT guidance, enhances the accuracy of drainage procedures. These technologies allow healthcare professionals to precisely locate the infected area, ensuring that drainage is performed effectively, minimizing the risk of complications, and promoting successful healing [71].

Debridement and Wound Care

In localized infections or wound breakdown, debridement plays a pivotal role. Debridement involves the removal of necrotic or nonviable tissue from the wound site. By eliminating tissue unlikely to heal or harbouring infection, debridement helps create an environment conducive to healthy tissue regeneration. This process serves several purposes. It reduces the bacterial load, as infectious agents often thrive in necrotic tissue. Additionally, it promotes angiogenesis, which is the formation of new blood vessels, and supports the growth of granulation tissue, a critical phase of wound healing. Proper wound care, including appropriate dressings, is vital for preventing further contamination, protecting the wound from external pathogens, and promoting successful healing [72]. Effective wound care addresses the immediate infection and helps mitigate potential complications from improperly managed wounds [72].

Supportive Care and Pain Management

While targeting the infection is crucial, managing postoperative infections extends beyond eradicating the infectious agents. Patients who experience postoperative infections often contend with pain, discomfort, and systemic symptoms. Therefore, comprehensive care involves addressing these issues through supportive measures. Adequate pain management is vital for patient comfort and well-being. Properly managed pain improves the patient’s experience and promotes mobility and faster recovery. Providing supportive care such as hydration and nutrition is essential alongside pain management. Maintaining proper fluid balance and ensuring adequate nourishment support the body's immune response and overall recovery process [73].

Addressing Complications

In addition to the potential complications mentioned earlier, postoperative infections can lead to other critical conditions, including bacteremia, septicemia, acute renal failure, and disseminated intravascular coagulation (DIC), all carrying a significant mortality risk. Bacteremia refers to bacteria in the bloodstream, septicemia denotes a severe systemic response to infection, acute renal failure indicates a sudden loss of kidney function, and DIC involves abnormal blood clotting throughout the body. These complications are grave and can lead to fatal outcomes if not promptly and appropriately managed. Managing postoperative infections and their associated complications demands specialized expertise and a multidisciplinary approach. It often requires collaboration among various medical specialists, such as infectious disease specialists, surgeons, and intensivists. This collaborative effort ensures comprehensive care that considers the infection and its impact on the body and any resulting complications [74].

Emerging trends and research

Innovations in Infection Prevention Techniques

In recent years, the landscape of infection prevention in gynaecological surgery has been marked by notable innovations, each aimed at mitigating postoperative infections. These advancements underscore the field's commitment to improving patient outcomes and revolutionizing how infections are managed in gynaecology [75].

One notable innovation involves the integration of antimicrobial-coated implants. Implantable medical devices, crucial in various gynaecological procedures, have been susceptible to postoperative infections due to the potential for bacterial colonization on their surfaces. Applying antimicrobial coatings to these implants holds great promise in reducing the risk of infections. These coatings release agents that actively inhibit bacterial growth, creating an inhospitable environment for pathogens. By curbing bacterial colonization, antimicrobial-coated implants have the potential to significantly diminish the occurrence of device-related infections, thereby enhancing patient safety and outcomes [76].

Furthermore, the emergence of minimally invasive techniques has transformed the paradigm of surgical approaches. Unlike traditional open surgeries, these approaches entail smaller incisions, reduced tissue trauma, and shorter recovery times. Significantly, they also correlate with a lower risk of infection. The decreased size of incisions minimizes the potential entry points for pathogens, while the reduced tissue manipulation decreases the overall bacterial load introduced into the body. Thus, the widespread adoption of minimally invasive techniques in gynaecological surgeries, coupled with the implementation of Enhanced Recovery After Surgery (ERAS) protocols, represents a substantial stride towards lowering infection risks and improving the overall patient experience [77].

Parallel to these developments, the field has emphasized advancing sterilization methods. Sterilizing surgical instruments and equipment is paramount in preventing infections from contaminated tools. The ongoing research into refining sterilization techniques addresses the challenges of complex instruments and sensitive materials. These efforts include exploring novel sterilization modalities, improving equipment design, and enhancing quality control processes. Enhancing sterilization methods makes the surgical environment more resistant to contamination, reinforcing the goal of reducing infection risks [43].

New Insights into Pathogenesis and Host Response

Advancements in microbiology and immunology have provided new insights into the interactions between pathogens and the host. Understanding the mechanisms by which pathogens evade the immune response and cause infections can lead to targeted interventions. Furthermore, insights into individual variations in immune responses may guide personalized treatment approaches [78].

Advances in Diagnostic Methods

The field of diagnostics has benefited from technological advancements, leading to more accurate and rapid identification of pathogens. Molecular techniques, such as polymerase chain reaction (PCR) and next-generation sequencing, enable the identification of pathogens from clinical samples with high sensitivity and specificity. Point-of-care testing is also gaining traction, allowing timely diagnosis and treatment [79].

Evolving Antibiotic Resistance Patterns

The emergence of antibiotic-resistant bacteria is a significant concern in managing postoperative infections. Monitoring and understanding evolving resistance patterns is crucial for selecting appropriate antibiotics. Developing novel antimicrobial agents, including bacteriophages and antimicrobial peptides, also offer potential alternatives to traditional antibiotics [80].

Future directions

Tailoring Prevention Strategies

The future of preventing postoperative infections in gynaecology lies in tailoring strategies to individual patients and surgical procedures. Advancements in predictive modelling and risk assessment can enable healthcare providers to identify patients at higher risk for infections and implement personalized preventive measures. Customized approaches may involve optimizing patients' immune status, adjusting antimicrobial prophylaxis regimens, and incorporating novel infection control technologies.

Personalized Treatment Approaches

As our understanding of individual variations in immune responses and microbial interactions deepens, personalized treatment approaches are on the horizon. Precision medicine may involve selecting antibiotics based on the patient's unique microbiome and immune profile, optimizing drug dosages, and targeting interventions to specific pathogenic mechanisms. This approach can improve treatment outcomes while minimizing the risk of antibiotic resistance.

Multidisciplinary Collaboration in Infection Management

Collaboration among various medical specialities is crucial for comprehensive infection management. Surgeons, infectious disease specialists, microbiologists, radiologists, and other healthcare professionals must work together to address the complex challenges of postoperative infections. Multidisciplinary teams can provide holistic care, leveraging expertise from different fields to tailor treatment plans and optimize patient outcomes.

Integration of Digital Health Technologies

Integrating digital health technologies, such as telemedicine, remote monitoring, and wearable devices, can enhance the management of postoperative infections. These tools enable healthcare providers to monitor patients' recovery progress, identify potential complications early, and provide timely interventions. Digital platforms also facilitate patient education, engagement, and follow-up, improving postoperative care.

## Conclusions

In conclusion, this review has delved into the intricate landscape of postoperative infections in gynaecology, shedding light on their various forms, such as surgical site infections, urinary tract infections, and pelvic inflammatory disease. By comprehending the significance of these infections, healthcare practitioners can actively work towards preventing their occurrence and effectively managing them when they arise. The significance of a holistic approach to managing postoperative infections must be considered. From meticulous preoperative assessments and tailored treatment strategies to vigilant postoperative care, every stage in the process is pivotal in reducing infection risks and ensuring favourable patient outcomes. The collaboration between surgical experts, infectious disease specialists, microbiologists, and other professionals is indispensable for navigating the intricacies of these challenges. Moreover, we emphasize the necessity of advancing research efforts, embracing emerging trends, and addressing evolving antibiotic resistance patterns. Additionally, fostering awareness among healthcare providers and patients about the critical importance of infection prevention and early detection remains essential in curbing the impact of these infections. This expedition from prevention to management of postoperative infections in gynaecology is characterized by continuous research advancements, innovative technological integration, and a resolute dedication to elevating patient care standards. By cultivating a more profound comprehension of these infections and fostering interdisciplinary cooperation, we edge closer to a future where the incidence of postoperative infections is minimized, patient outcomes are optimized, and the overall quality of healthcare is elevated.
